# Gate-Tunable Single Ag–rGO FET Platform for Room-Temperature Trigas Detection with a CMOS-Compatible PWM Readout Interface

**DOI:** 10.1021/acsomega.6c05753

**Published:** 2026-10-10

**Authors:** Mayank Kohli, Nimisha, Swapnil Sharma, Santanab Majumder, Gourab Dutta, Tarun Kanti Bhattacharyya

**Affiliations:** † Department of Electronics and Electrical Communication Engineering, 30133 Indian Institute of Technology, Kharagpur 721302, India; ‡ Manipal School of Information Sciences, Manipal 576104, India

## Abstract

Room-temperature gas sensing requires high sensitivity and selectivity, which FET-based architectures achieve through gate-controlled modulation of surface potential and carrier transport, enabling tunable and efficient chemical transduction. In this work, we introduce a smart trigas sensor capable of selectively detecting three target gases—acetone, formaldehyde, and ammonia at room temperature using a single FET by simply tuning the gate voltage. By leveraging gate-induced modulation, this study demonstrates dynamic control over the interaction between gas molecules and the silver-decorated Reduced Graphene Oxide (Ag-rGO) channel-based gas FET sensor, enabling the individual and selective detection of three distinct gases using the single device. Furthermore, the proposed platform demonstrates distinct cross-sensitivity characteristics, wherein gate-bias modulation enables selective discrimination between formaldehyde, acetone, and ammonia under mixed-gas environments. The device exhibits high detection capability with experimentally estimated limits of detection (LOD) of 158 ppb for acetone, 31 ppb for formaldehyde, and 36 ppb for ammonia, highlighting its potential for low-concentration, room-temperature multigas sensing applications. Beyond material and device-level innovation, a TSMC 180 nm 3.3 V CMOS readout circuit is designed and simulated in Cadence Virtuoso, featuring analogue front-end design for interfacing the proposed gas sensor with a low-power embedded system via PWM signals. The proposed multigas sensor platform and the designed and simulated readout circuit establish a compact and low-power sensing framework, providing a foundation for future studies on breath biomarker analysis and disease-detection applications.

## Graphical abstract

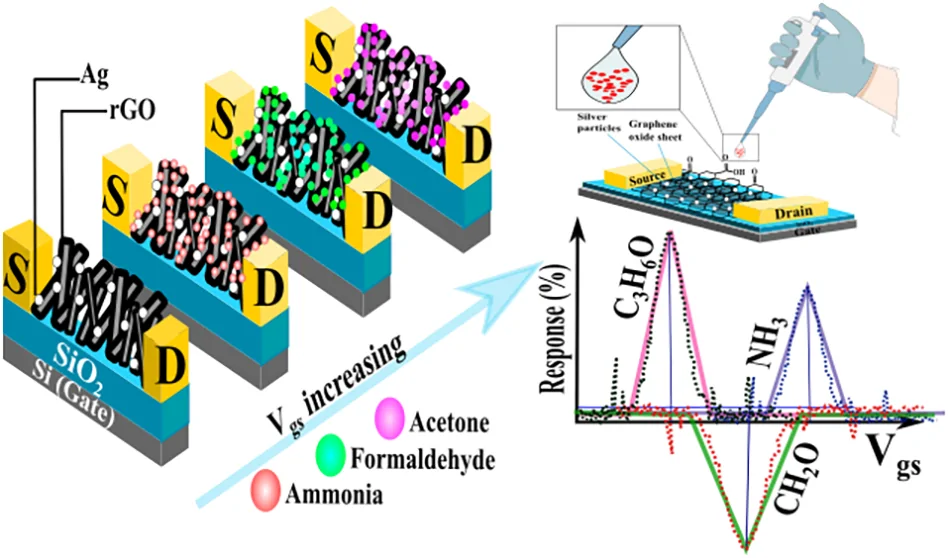

## Introduction

Many industrial, biomedical, and environmental systems rely on high-fidelity gas monitoring for real-time regulation and hazard mitigation, spanning applications such as domestic leak-detection systems, medical diagnostic instrumentation, environmental compliance monitoring, and chemical-plant process control.^1–3^ Among various sensing modalities, volatile organic compound (VOC) detectors have gained prominence due to their relevance in early, noninvasive disease detection.^2,4^ With increasing global healthcare demands, interdisciplinary domains such as theranostics have accelerated the development of advanced gas sensors, key performance parameters such as accuracy, selectivity, sensitivity, and response time are strongly governed by the choice of sensing materials.^1^ Exhaled human breath contains a broad range of VOCs and other gases such as formaldehyde, ammonia, acetone, methane, and toluene,^3^ whose concentration patterns are linked to specific physiological conditions. For instance, ammonia (NH_3_) correlates with renal dysfunction,^4^ acetone (C_3_H_6_O) with metabolic irregularities in diabetes,^5^ and formaldehyde (HCHO) with lung-cancer-associated abnormalities.^2^

Conventional breath analysis techniques such as gas chromatography coupled with mass spectrometry (GC-MS), selected-ion flow-tube mass spectrometry (SIFT-MS), and proton-transfer-reaction mass spectrometry (PTR-MS) have been widely employed for noninvasive diagnosis by detecting various components in exhaled breath.^6^ However, despite their accuracy, wide scale usability of these techniques are limited due to the high cost, operational complexity, and bulky instrumentation, which restrict their use primarily to clinical or laboratory settings. As a compact alternative, a field-effect transistor (FET) integrated with an active sensing channel material, enabling bias-controlled transduction with enhanced sensitivity. The active FET architecture allows precise gate-voltage modulation of channel conductivity and carrier concentration, thereby improving the responsiveness and selectivity of the sensing layer toward target gases. Additionally, catalytic nanoparticles, including Pt, Pd, and Au, facilitate selective chemical interactions with target gas molecules, inducing variations in the electrical resistance and conductivity of the sensing material.^7^ These interactions modulate the channel conductance of the device, thereby causing measurable changes in the source-drain current. Precise monitoring of these current fluctuations enables the identification and quantification of the specific gas species present.

Recent advancements aim to integrate individual gas sensors into unified arrays, each tailored for selectivity toward specific gases. These arrays enable simultaneous multigas detection through distinct electrical responses from gas-specific chemical interactions. The incorporation of IoT components including microcontrollers, Wi-Fi modules, and ADCs interface facilitates real time transmission of breath data to smartphones, enabling continuous monitoring.^8^ Machine learning has improved gas classification,^9^ but its implementation typically requires substantial preprocessing feature extraction, normalization, and model training along with large labeled data sets (often 10–12 times the number of feature parameters), leading to considerable computational and training overhead.

In this work, silver-decorated reduced graphene oxide (Ag-rGO) has been adopted as the sensing channel material for the FET, which is exposed to different gases (acetone, ammonia and formaldehyde). The large surface area and porosity of Ag-rGO facilitate efficient gas adsorption, simple fabrication, and sensor reusability. By employing gate voltage modulation, the same sensor demonstrates enhanced sensitivity and effective discrimination among multiple gases at varying concentrations. While the present work is limited to controlled laboratory conditions, it demonstrates the feasibility of selective multigas sensing using a single FET and lays the groundwork for future studies involving realistic breath samples and disease-specific VOC analysis.

The interfacing and readout circuitry play a pivotal role in translating raw transducer outputs into robust, system-level signals. In this work, a dedicated TSMC 180 nm, 3.3 V CMOS analogue front-end (AFE) readout circuit is designed and simulated in Cadence Virtuoso. The circuit integrates bias generation, logarithmic current-to-voltage conversion, and PWM-based signal encoding, enabling reliable extraction of sensor responses even at low current levels while maintaining immunity to noise. Overall, this work presents a multigas sensing platform together with a designed and simulated interfacing readout circuit, serving as a foundational step toward future breath biomarker analysis and disease-detection systems. Although the present study is limited to controlled gas environments, the proposed sensor–circuit framework demonstrates the feasibility of compact, low-power, and scalable multigas sensing, providing a strong foundation for future validation under realistic breath conditions.

## Fabrication

The FET-based gas sensor was fabricated on a heavily boron-doped p-type Si ⟨100⟩ substrate (2-in., 0.001–0.005 Ω·cm, 250–300 μm thick) with a 150 nm thermally grown SiO_2_ layer serving as the gate dielectric, where the silicon substrate also functioned as the back gate for the FET.

A 20 nm Ti layer and 180 nm Au layer were deposited via DC sputtering and then patterned to form interdigitated electrodes (IDEs) defined by standard photolithography, with an effective transistor width of 12,000 μm and a channel length of 200 μm. A solution of silver-decorated reduced graphene oxide (Ag-rGO) was drop-cast onto the channel region and dried at 60 °C to ensure uniform film coverage and contact. The fabricated device is shown in Figure 1a. The sensor operates on the interaction between gas analytes and the Ag-rGO sensing channel. Adsorption of gases induces charge transfer, modulating the channel’s conductivity. Gate voltage tuning enables selective adsorption by altering surface charge, allowing discrimination between gases based on their interaction enthalpies, as illustrated in Figure 1b. Figure 1c illustrates the schematic representation of selective gas adsorption achieved through gate-voltage tuning, wherein modulation of the surface charge affinity enables preferential interaction with different target gas molecules.

**Figure 1:**
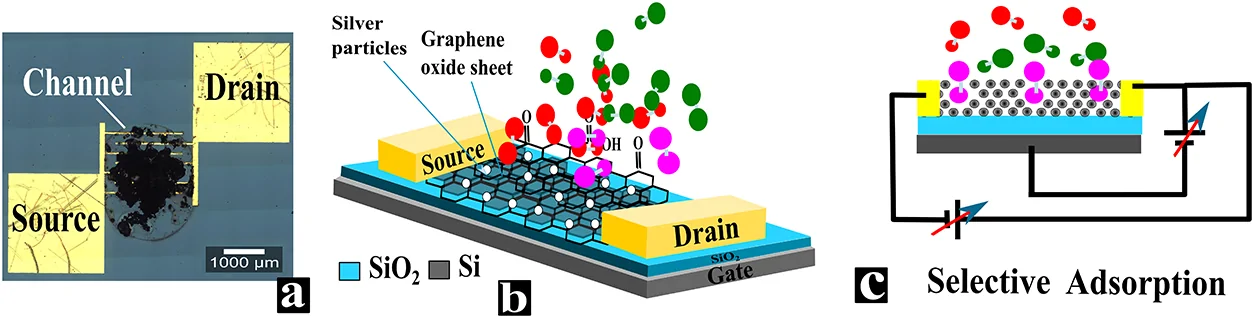
(a) Optical image of the fabricated and drop-casted Ag–rGO FET device, (b) Schematic diagram of the FET configuration provided as a charged surface for specific adsorption of particular analyte, (c) Schematic diagram of selective gas adsorption achieved through gate-voltage tuning.

## Material Synthesis and Characterization

Graphene oxide (GO) was synthesized from high-purity graphite powder using a modified Hummer’s method,^10^ shown in Figure S1. To prepare silver-decorated reduced graphene oxide (Ag-rGO), 20 mL of the previously synthesized rGO dispersion was diluted with 10 mL of deionized water. To this solution, 100 μL of silver nitrate (AgNO_3_) was added, followed by the gradual addition of 0.5 g of sodium borohydride (NaBH_4_) as the reducing agent. The resulting mixture was stirred vigorously at room temperature for 30 min to facilitate the *in situ* reduction of Ag^+^ ions and their uniform decoration onto the rGO surface. The formation of Ag nanoparticles on the rGO sheets was indicated by texture change of the solution. For device fabrication, 1 μL of the Ag-rGO (2 mg/mL) solution was drop-cast onto the IDE and allowed to dry at 60^*o*^C, forming the sensing layer.

In Figure 2a, the Raman spectra of both rGO and Ag–rGO samples are presented. The spectra display the characteristic D and G bands, observed at 1349 and 1581 cm^–1^ for rGO, and at 1353.5 and 1567 cm^–1^ for Ag–rGO, respectively. The intensity ratio of D and G bands was estimated to be 1.005 for rGO and 0.993 for Ag–rGO, indicating a marginal reduction in structural disorder upon silver decoration. In Ag–rGO, an enhanced G band intensity was observed, likely due to the interaction of silver nanoparticles with the graphene lattice, resulting in surface-enhanced Raman scattering (SERS).

**Figure 2:**
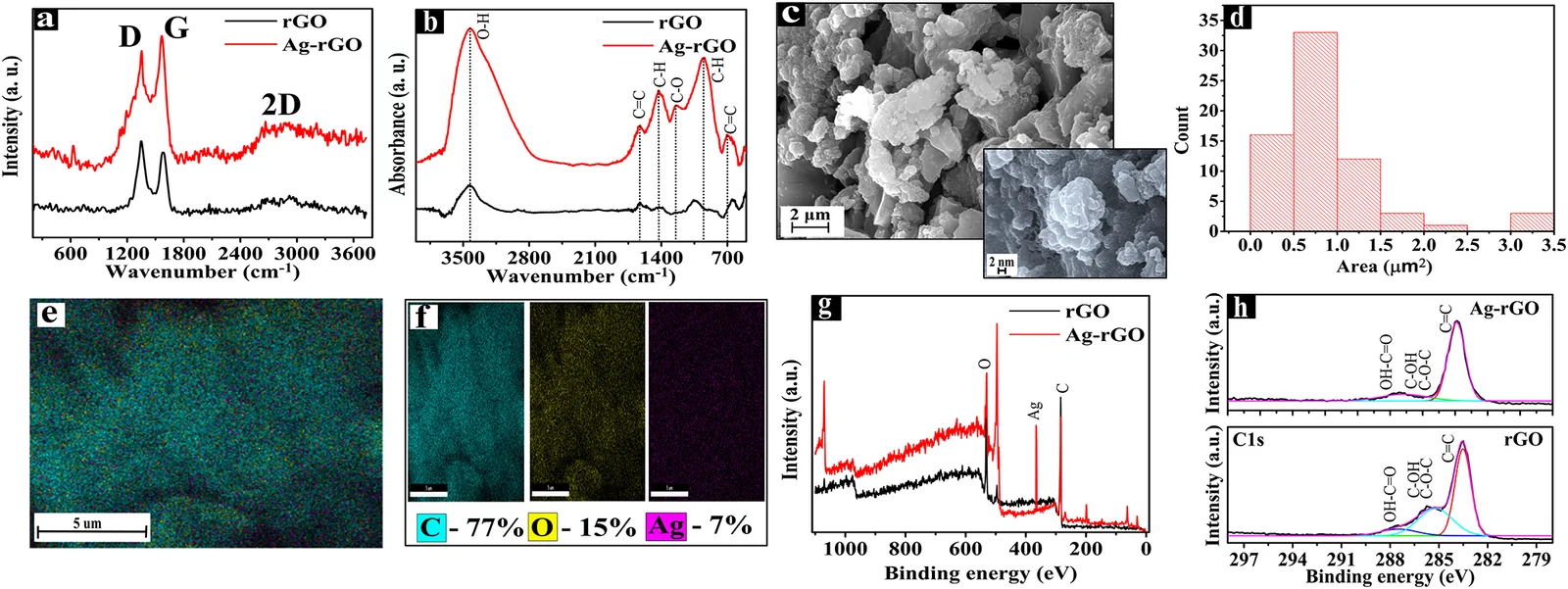
Material characterization (a) Raman spectra of rGO and Ag–rGO, (b) FTIR spectra of rGO and Ag–rGO, (c) SEM image with magnified view of Ag–rGO nanosheets, (d) SEM-based surface area distribution profile, (e) EDAX elemental profile of Ag–rGO, (f) elemental mapping of carbon, oxygen, and silver, (g) XPS survey spectra, and (h) high-resolution XPS spectra of C 1s for rGO and Ag–rGO.

Figure 2b illustrates the Fourier Transform Infrared (FTIR) spectra of pristine rGO and Ag-decorated rGO (Ag–rGO) nanocomposites. The FTIR spectrum of rGO exhibits characteristic absorption bands corresponding to −OH stretching vibrations at 3426 cm^–1^, C=C skeletal vibrations (υ_C=C_) near 700 cm^–1^, C–H stretching and bending modes (υ_C–H_) at 1428 and 951 cm^–1^, C–O stretching (υ_C–O_) at 1241 cm^–1^, and C=O stretching (υ_C=O_) at 1725 cm^–1^, confirming the presence of residual oxygen-containing functional groups. Upon incorporation of silver nanoparticles, either through intercalation between the graphitic layers or deposition onto the rGO surface, the overall spectral profile of Ag–rGO remains comparable to that of rGO. However, a notable enhancement in the intensity of the characteristic bands is observed, indicating improved interaction and electronic coupling between the Ag nanoparticles and the rGO matrix.^11^

Figure 2c presents the Field Emission Scanning Electron Microscopy (FESEM) micrograph of Ag–rGO, revealing its characteristic flake-like morphology. The well-defined, layered flakes provide a high surface area, which is beneficial for enhancing the physisorption of target gas species. The magnified view (inset of Figure 2c) highlights the uniform distribution and surface texture of the Ag-decorated rGO sheets. Image analysis, as shown in Figure 2d, indicates that the average area of the microstructural flakes lies in the range of 0.5–1 μm^2^, confirming a consistent microstructure conducive to efficient surface interaction.

Elemental analysis via Energy Dispersive X-ray Analysis (EDAX), shown in Figure 2e, confirms the presence of approximately 15% oxygen, 77% carbon, and 7% silver, validating the successful surface decoration of rGO with Ag nanoparticles. The EDAX spectrum further indicates the compositional uniformity of the synthesized Ag–rGO. Figure 2f presents the corresponding elemental mapping, illustrating the spatial distribution of carbon, oxygen, and silver elements, which confirms homogeneous dispersion of Ag across the rGO surface. X-ray photoelectron spectroscopy (XPS) analysis was performed to investigate the surface chemical composition of rGO and Ag–rGO samples. The XPS survey spectrum (Figure 2g) reveals distinct binding energy peaks corresponding to C 1s (285.0 eV), O 1s (531.5 eV), and Ag 3d (368.0 eV–374.0 eV), confirming the successful incorporation of silver in the Ag–rGO nanocomposite. The high-resolution C 1s spectrum of rGO (Figure 2h) displays three characteristic peaks centered at approximately 284.0, 285.3, and 288.2 eV, attributed to C=C, C–O, and C=O bonds, respectively, confirming the partial reduction of graphene oxide. In comparison, the C 1s spectrum of Ag–rGO exhibits peaks at 284.0 and 287.9 eV corresponding to C=C and C=O bonds, respectively, while the intensity of the C–O component is notably diminished, indicating a further reduction of oxygen-containing functional groups upon Ag incorporation.

## Results and Discussion

The electrical response of the Ag-rGO FET sensor was systematically evaluated both in ambient conditions and under exposure to acetone, formaldehyde, and ammonia once at a time at room temperature. Figure 3 shows the sensor response as a function of gate voltage for individual gas exposures. The observed behavior of FET characteristics confirms that Ag–rGO forms a p-type semiconducting channel, as prominent from the nature of the transfer characteristics. Ag decoration induces p-type behavior in rGO FETs primarily due to these factors: (i) residual oxygen functional groups in rGO act as electron-withdrawing sites, leading to intrinsic hole conduction; (ii) work-function mismatch between Ag and rGO facilitates electron transfer from rGO to Ag, shifting the Fermi level toward hole dominance, the combination of these effects produces transfer curves consistent with p-type FET behavior.^12^ As evident from Figure 3, each gas produced a distinct response profile with gate modulation of gate voltage, for acetone, the current increases without any notable change in threshold voltage (*V*_th_); for formaldehyde, *V*_th_ shifts to more negative values without a substantial change in current; and for ammonia, a significant increase in current is observed along with a positive shift in *V*_th_.

**Figure 3:**
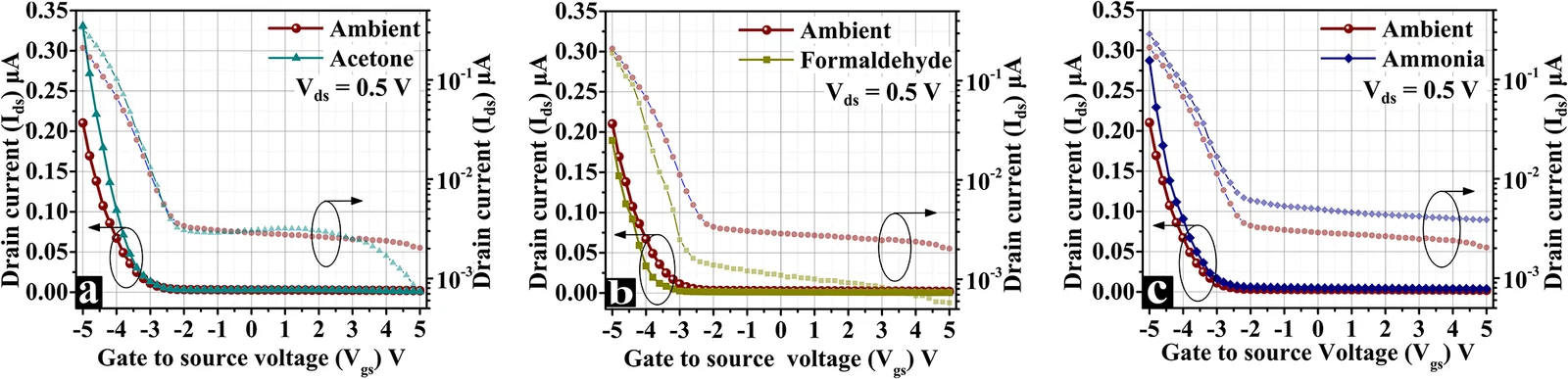
Transfer characteristics of the sensor with 2 ppm and without any gas exposure: (a) Acetone (C_3_H_6_O), (b) Formaldehyde (HCHO), and (c) Ammonia (NH_3_), at room temperature.

Upon exposure to target gases, the electrical conductivity of the Ag–rGO channel is perturbed depending on the nature of the gas. This modulation in charge carrier concentration and mobility arises from the interaction of gas molecules with the channel surface, thereby altering the electrical response of the device. The sensing mechanism can also be explained from the band bending at the Ag–rGO interface, which is significantly influenced by the difference in work functions between Ag nanoparticles and rGO. Before gas exposure, oxygen molecules from the ambient air are adsorbed at defect sites in the rGO, which are also responsible for introducing a finite bandgap.^13,14^

The band alignment between Ag nanoparticles and rGO results in the formation of a Schottky barrier, governed by their respective work functions. Since the work function of Ag (ϕ_Ag_ = 4.2–4.5 eV) is lower than that of rGO (ϕ_rGO_ ≈ 5.1 eV), electrons flow from Ag to rGO until equilibrium is established, leading to hole annihilation in the rGO (Figure 4).^15^ Additionally, the defect states in rGO give rise to a charge neutrality level, which lies below the Fermi level.^16^ Electron transfer from Ag nanoparticles occurs predominantly to this charge neutrality level, rather than directly to the Fermi level of rGO, further modulating the interface energetics. The built-in potential, which inhibits charge transfer from rGO to Ag nanoparticles, can be modulated through exposure to reducing or oxidizing gases, respectively. Reducing gases such as ammonia (NH_3_) interact to the Ag nanoparticles, raising their Fermi level and facilitating charge transfer back into rGO. This decreases the built-in potential resulting enhanced electron injection, resulting in a shift in threshold voltage.^16^ Furthermore, the carrier mobility is also affected due to this interfacial interaction, as depicted. Through this mechanism, Ag nanoparticles serve as electron mediators and energy-altering interface modifiers, thereby significantly enhancing the sensitivity and selectivity of the Ag–rGO based FET sensor toward various distinct gas analytes. The sensing material serves mainly as a platform for gas accumulation, whereas the magnitude and direction of the surface potential shift are determined by the redox characteristics of the adsorbed species, whether they act as oxidizing or reducing agents.

**Figure 4:**
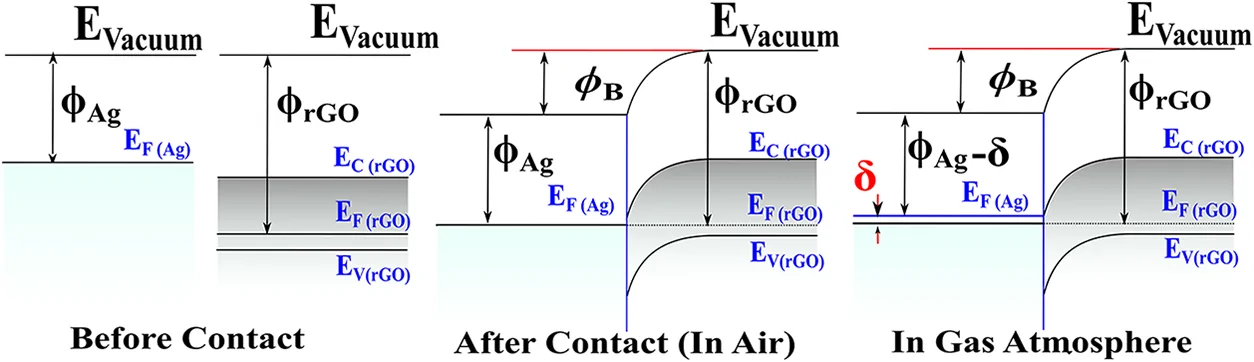
Energy band diagram for Ag–rGO composite, in air and in gas atmosphere. [ϕ_Ag_ and ϕ_rGO_ represent the work function of Ag and the electron affinity of rGO, respectively, whereas ϕ_B_ denotes the built-in potential that is modulated by δ upon exposure to gases.].

Figure 5 illustrates the response behavior of the Ag-rGO FET sensor when exposed to individual and mixed gases, with the response defined as

1 $$response\;\left(\%\right)=\frac{I_{with\;gas}-I_{without\;gas}}{I_{without\;gas}}\times 100$$

**Figure 5:**
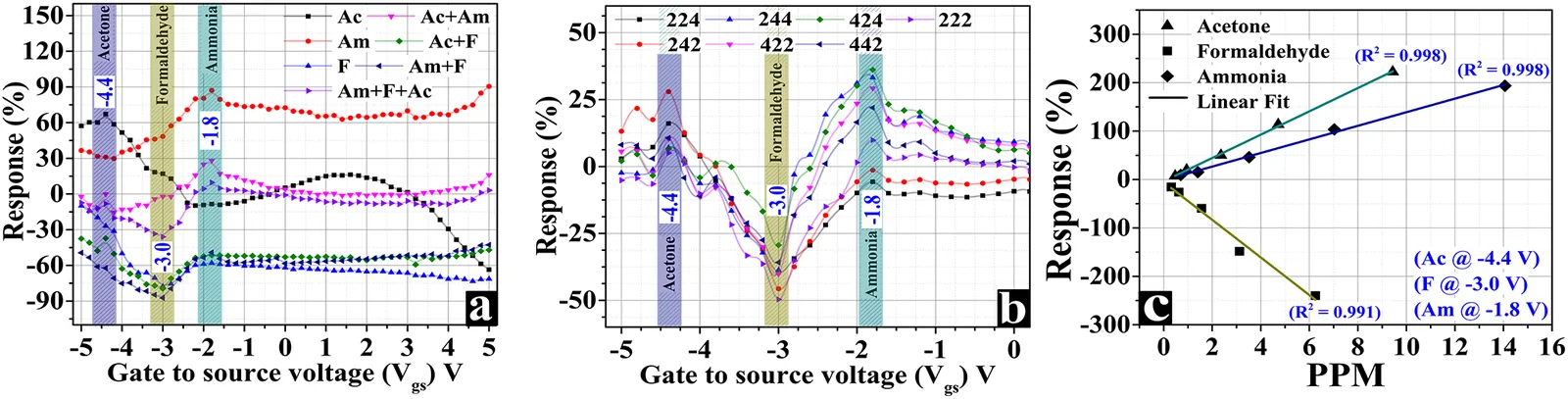
(a) Sensor response of Device 1 as a function of gate voltage under exposure to individual gases and mixed gas environments at room temperature, where F, Ac, and Am denote formaldehyde, acetone, and ammonia, respectively at 2 ppm for 30s. (b) Response characteristics of Device 2 versus gate voltage during simultaneous exposure to all three gases at varying concentration combinations. The notation “444” represents F, Ac, and Am each at 4 ppm, while “422” represents formaldehyde at 4 ppm with acetone and ammonia maintained at 2 ppm. (c) Response variation of Device 3 with changing gas concentrations, illustrating the concentration-dependent sensitivity characteristics of the sensor.

Notably, each gas induces a maximum/minimum response (peak or dip) at a specific gate voltage (acetone at −4.4 V, formaldehyde at −3.0 V, and ammonia at −1.8 V). This gate-tuned selectivity is attributed to the unique enthalpy of adsorption required by each gas molecule.^17,18^ As shown in Figure 3, ammonia and acetone exposure increase channel conductivity, while formaldehyde reduces it, consistent with the respective peak and dip response signatures observed in Figure 5a, irrespective of mixing of different gases. These characteristic responses enable selective identification of gases in mixtures, where each gas maintains its distinct electrical behavior (peak or dip) at its corresponding adsorption gate voltage, even when exposed to simultaneously. To test this another device (device 2), fabricated on same wafer was used. The response of the device 2 under different combinations of gas mixture is shown in Figure 5b for the entire sweep of the gate voltage. This response clearly indicates that the corresponding peak/dip positions remained the same, and the presence of individual gases can be identified from the response peak/dip at that particular gate voltage. The peak or dip position corresponding to each analyte in the gas mixture remains identical to the observed under individual gas exposure, and this consistency is maintained across all devices fabricated on the same wafer. The observed variation in response percentage between device 1 and 2 is attributed to channel layer nonuniformity across devices, originating from the drop–casting method used for channel formation. This highlights the need for further optimization, including the adoption of alternative channel deposition techniques in place of drop casting. Figure 5c also demonstrates a linear relationship between the sensor response and the gas concentration for acetone, formaldehyde, and ammonia. The sensing response of the Ag-rGO TFT toward an individual gas exhibits an approximately linear dependence on gas concentration within the investigated concentration range. Therefore, the response for each analyte can be expressed as-

2 $$Y_{i}=m_{i}X_{i}+c_{i}$$

where *Y*_*i*_ is the measured sensor response, *X*_*i*_ is the concentration of the *i*th gas, *m*_*i*_ is the sensitivity obtained from the calibration curve, and *c*_*i*_ represents the baseline offset. In this work, *i* = 1, 2, and 3 correspond to ammonia (NH_3_), formaldehyde (HCHO), and acetone (CH_3_COCH_3_), respectively.

When multiple gases are simultaneously present, the overall sensor response is influenced by the individual adsorption affinity and adsorption enthalpy of each gas molecule on the Ag-rGO sensing layer. Assuming that the contributions of the gases are additive, the sensor response (Y) at a particular gate voltage (*V*_gs_) can be expressed as-

3 $$Y\left(V_{gs}\right)=a_{1}X_{1}+a_{2}X_{2}+a_{3}X_{3}+a_{12}X_{1}X_{2}+a_{13}X_{1}X_{3}+a_{23}X_{2}X_{3}+D$$

where (*Y*(*V*_*gs*_)) denotes the measured sensor response at the selected gate voltage, *X*_1_, *X*_2_, and *X*_3_ represent the concentrations of ammonia (NH_3_), formaldehyde (HCHO), and acetone (CH_3_COCH_3_), respectively. The coefficients *a*_1_, *a*_2_, and *a*_3_ correspond to the individual sensitivities of the sensor toward each gas, whereas a_12_, a_13_, and a_23_ quantify the pairwise interaction effects arising from the simultaneous adsorption of two different gas species on the Ag-rGO sensing layer. The constant D represents the baseline response of the device. The performance of the proposed quantification model is summarized in Table 1, which presents the actual and predicted concentrations, along with the corresponding prediction errors, for gas mixtures. As shown in the table, the proposed model accurately predicts the concentrations for most gas mixtures; however, relatively larger errors are observed for a few cases. These deviations are primarily attributed to the simplified interaction model, which may not fully capture the complex adsorption behavior and cross-sensitivity effects among multiple gases. The prediction accuracy can be further improved by developing a more comprehensive multigas response model that incorporates higher-order interaction effects or more representative calibration data.

**Table 1:** Actual and Predicted Concentrations with Prediction Errors

	**measured response (** *Y* **)**			**actual conc.**	**predicted conc.**	
**sample**	–4.4 V	–3.0 V	–1.8 V	(*X*_1_, *X*_2_, *X*_3_)	(*X*_1_, *X*_2_, *X*_3_)	**Error**
1	16.40	–39.50	–5.62	(2, 2, 4)	(2.000, 2.000, 4.000)	(0.000, 0.000, 0.000)
2	28.10	–45.70	–1.32	(2, 4, 2)	(2.000, 4.000, 2.000)	(0.000, 0.000, 0.000)
3	7.00	–38.70	33.00	(2, 4, 4)	(2.155, 4.050, 3.756)	(+0.155, +0.050, −0.244)
4	10.43	–40.00	29.25	(4, 2, 2)	(1.846, 4.104, 3.876)	(−2.154, +2.104, +1.876)
5	6.63	–29.64	36.43	(4, 2, 4)	(4.000, 2.000, 4.000)	(0.000, 0.000, 0.000)
6	10.63	–35.38	21.94	(4, 4, 2)	(3.424, 1.843, 3.468)	(−0.576, −2.157, +1.468)
7	5.00	–49.54	10.00	(2, 2, 2)	(2.000, 2.000, 2.000)	(0.000, 0.000, 0.000)

The effects of relative humidity, device-to-device variation, and prolonged ambient exposure on the sensor performance were systematically evaluated, as shown in Figure 6. The dynamic variation of relative humidity at (*V*_gs_ = 0) V is shown in Figure 6a. The transfer characteristics exhibit negligible changes under different relative humidity (RH) conditions (Figure 6b), indicating that the Ag-rGO FET sensor is largely insensitive to humidity-induced interference. The long-term stability of the three TFT sensors was assessed over 9 days, as shown in Figure 6c, confirming stable electrical characteristics during ambient storage. Figure 6d illustrates the device-to-device variation, indicating good reproducibility of the fabrication process. The surface profilometry of different drop-cast Ag-rGO devices is shown in Figure 6e, which verifies the uniformity of the deposited films and confirms an electrode step height of approximately 200 nm. Furthermore, the sensor response over the 9-day evaluation period (Figure 6f) exhibits consistent peak and dip positions with minimal signal degradation, demonstrating excellent temporal stability and repeatability of the sensing platform.

**Figure 6:**
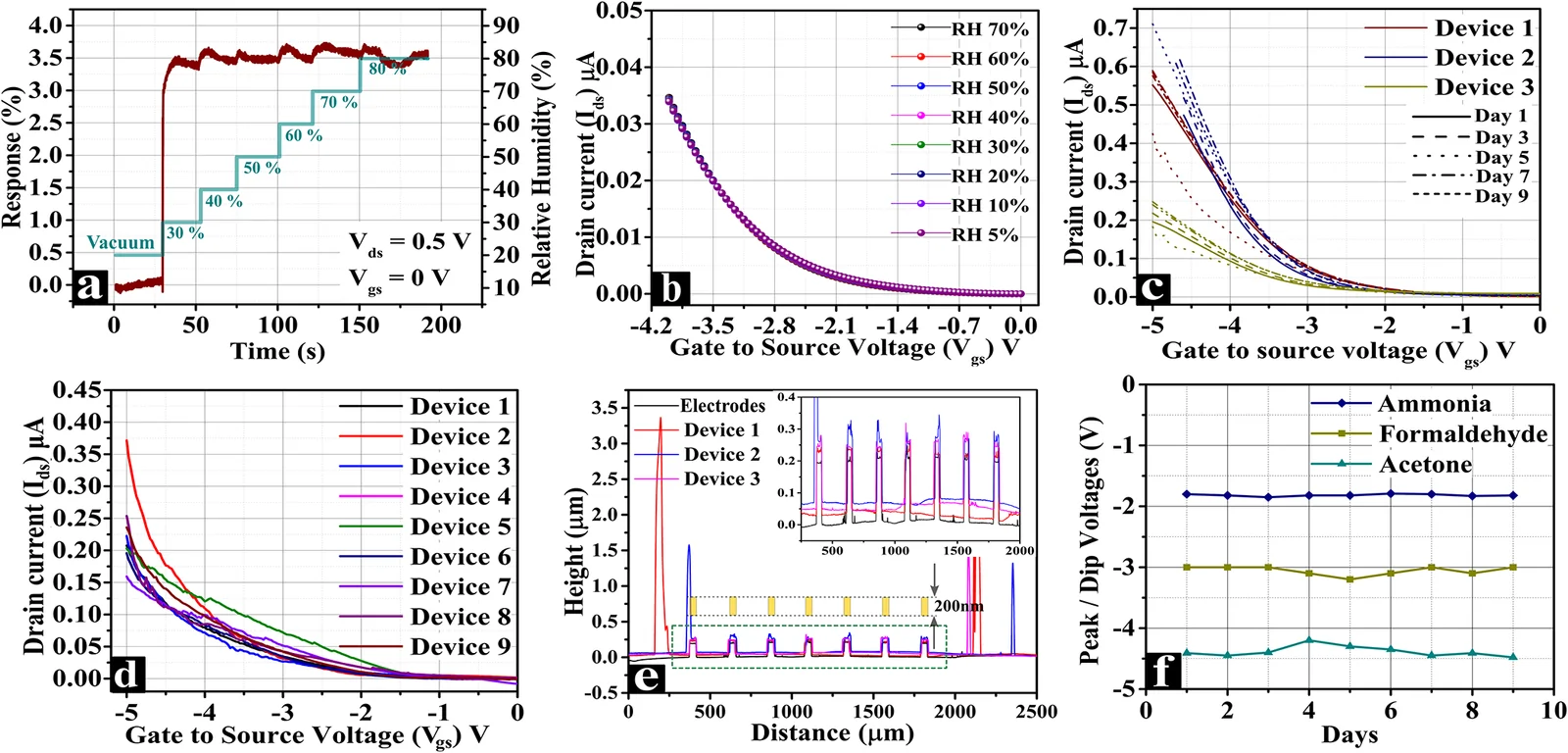
(a) Dynamic humidity variation as a function of time (*V*_gs_ = 0 V). (b) Transfer characteristics of the Ag-rGO FET under different relative humidity (RH) conditions. (c) Long-term stability of the Ag-rGO FET sensor measured over 9 days. (d) Device-to-device variation of the fabricated Ag-rGO FET sensors. (e) Surface profile analysis of Ag-rGO films deposited by drop-casting on different devices. (f) Sensor performance of the fabricated devices over time.

The selectivity analysis (Figure S2) demonstrates a pronounced response toward ammonia, formaldehyde, and acetone compared to other tested analytes, confirming preferential interaction with the target analytes. The transient dynamic response under optimized gate bias conditions is presented in Figure S3. The extracted adsorption time constants are 3.63 s for ammonia at *V*_gs_ = −1.8 V, 4.91 s for formaldehyde at *V*_gs_ = −3.0 V, and 1.52 s for acetone at *V*_gs_ = −4.4 V, measured at *V*_ds_ = 0.5 V (Figure S4). Statistical study across multiple devices is summarized in Figure S5, demonstrating consistent response magnitude with acceptable device-to-device variation for the targeted gases.

As illustrated in Figure 5, the qualitative identification of different gas species can be inferred by inspecting the characteristic peaks in the sensor’s percentage response. However, for reliable quantitative processing subsequent interfacing with embedded microcontrollers, it is essential to accurately capture the ratio of currents corresponding to each instantaneous *V*_gs_ in real time and transmit the signal. An interfacing circuit is designed and simulated in the Cadence Virtuoso environment at the 180 nm technology node using 3.3 V high-voltage transistors for interfacing with embedded systems, producing a pulse-width-modulated (PWM) output for improved noise immunity compared to transmitting the signal in analogue form.

To implement the required division of sensor currents, a logarithmic signal-conditioning circuit followed by a subtractor stage is employed, as shown in Figure 7a. In this architecture, the instantaneous current of the active gas-exposed sensor is mathematically divided by the reference sensor current (obtained from a device shielded from gas exposure). The subtractor unit is realized using a PMOS-driver operational transconductance amplifier (OTA) configured in resistive feedback. A reference voltage of 1.65 V is applied to the *V*_ocm_ pin of the subtractor (Figure 7a) to establish a fixed output common-mode voltage of *V*_dd_/2. Consequently, the output node *V*_out_ effectively represents the logarithm of ratio *I*_gas_/*I*_ref_, centered around the *V*_dd_/2 V common-mode point. The bias voltage *V*_bias_ is applied to establish the drain-to-source voltage *V*_ds_ of the sensors, ensuring stable operation and consistent current generation during gas exposure.

**Figure 7:**
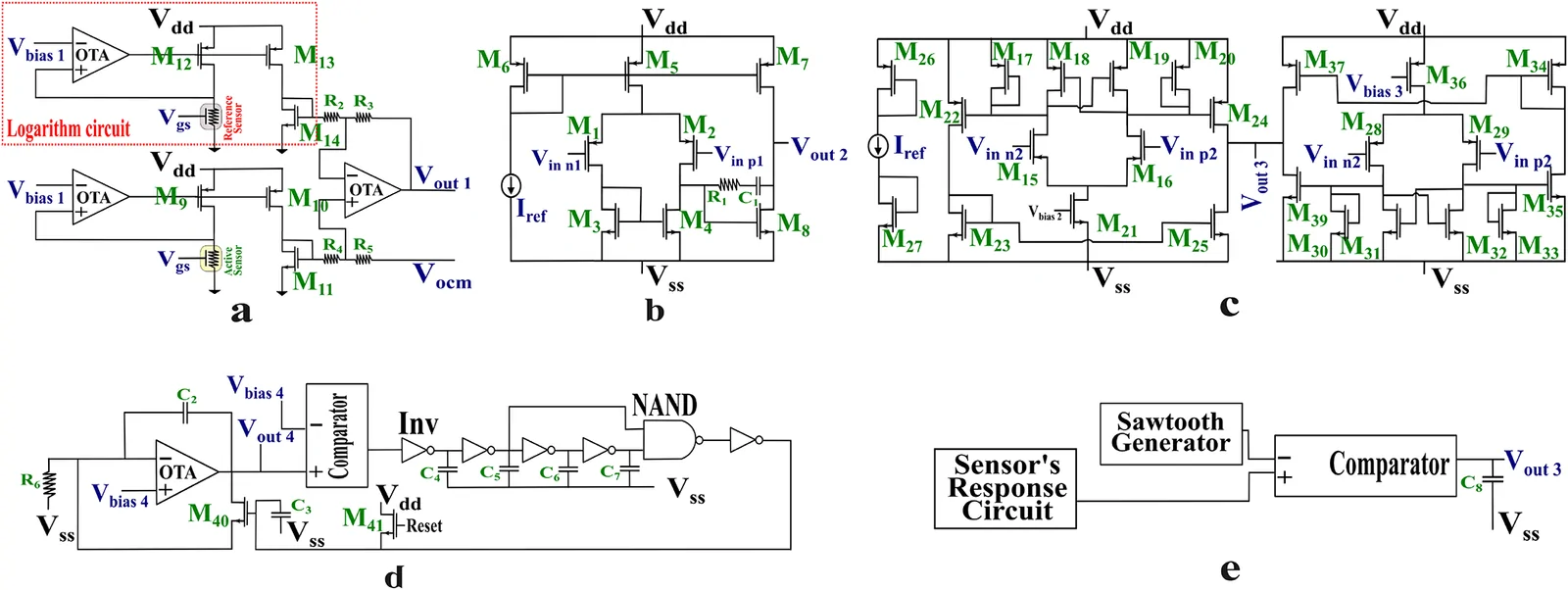
Interfacing circuit: (a) Sensor’s response circuit realize the current-division logic *I*_with gas_/*I*_without gas_, (b) a PMOS-driven two-stage operational transconductance amplifier (OTA) for analogue signal conditioning, (c) the comparator circuit, (d) the sawtooth generator, and (e) the overall block diagram of the complete sensing interface circuitry.

To ensure accurate logarithmic behavior, MOSFETs *M*_11_ and *M*_14_ within the log-domain processing block are biased in the subthreshold region (Figure 7a). The PMOS two-stage driver OTA used in the subtractor is designed to provide a high open-loop gain of 85 dB and a phase margin of approximately 80°, ensuring both precision and stability under closed-loop operation, as depicted in Figure 7b.

For interfacing with low power embedded systems, a pulse-width-modulation (PWM)-based conversion scheme is implemented to enable robust and noise-immune transmission of the processed information. The duty cycle of the PWM signal varies proportionally to the ratio *I*_gas_/*I*_ref_, thereby encoding the gas-dependent sensing information into a time-domain digital format suitable for low-power microcontroller-based readout. To realize the PWM encoding, a sawtooth generator circuit is implemented (shown in Figure 7d).^19^ Finally, a comparator circuit (shown in Figure 7c)^20^ is used to compare the output of the sensor’s divider stage with the sawtooth waveform (derived from the sawtooth generator) to produce the PWM signal.

The circuit shown in Figure 7 provides the required biasing for the gas sensors through the applied drain-source bias *V*_bias_, while simultaneously mirroring the sensor current with a scaling factor of 0.1. This mirrored current is fed into a MOSFET device designed with a significantly large *W*/*L* ratio, ensuring that the transistor predominantly operates in the cutoff or subthreshold region. Operation in this regime enforces an exponential current–voltage relationship, enabling the circuit to generate a logarithmic representation of the sensor current. This behavior is validated in Figure 8a, where the input current is swept from 1 nA to 1 mA. With the *x*-axis plotted on a logarithmic scale, the resulting voltage output characteristic obtained from the logarithmic circuit exhibits near-linear behavior, confirming that the output voltage is proportional to the logarithm of the input current.

**Figure 8:**
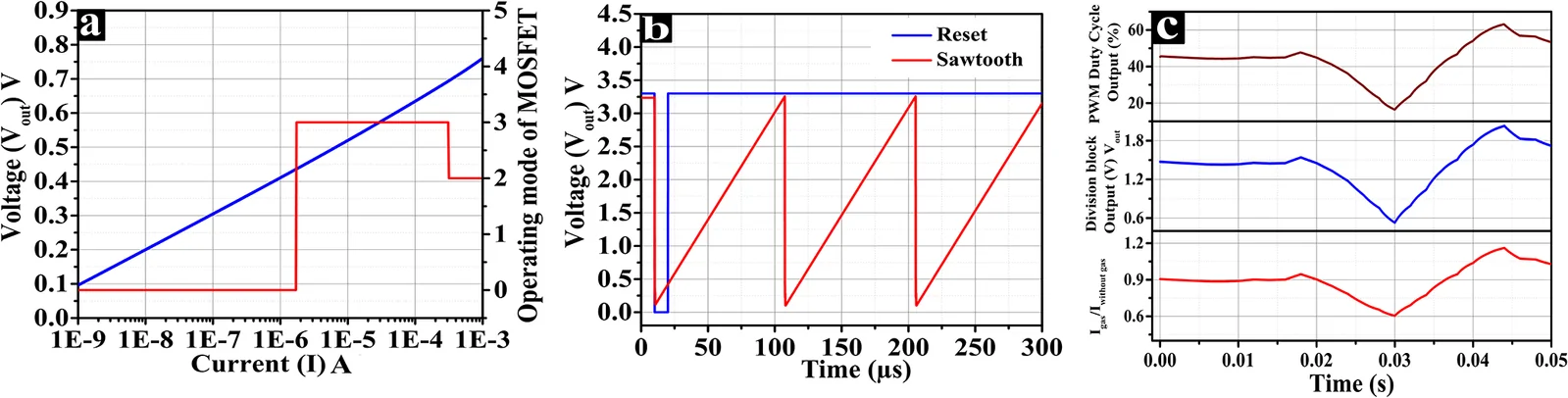
(a) Input–output I–V characteristics of the logarithmic transduction circuit presented in Figure 7a, (b) output waveform of the implemented sawtooth generator, and (c) comparative analysis of the PWM duty cycle (circuit shown in Figure 7e) with the division-circuit output (circuit shown in Figure 7a) and the theoretical ratio *I*_gas_/*I*_without gas_.

Figure 8a also includes an operation-region, which identifies the instantaneous region of operation of the MOSFET. For currents in the range of 1 nA to approximately 325 μA, the device transitions between region codes 0 and 3, corresponding to cutoff and subthreshold operation, respectively. Beyond 325 μA, the region code changes to 2, indicating that the MOSFET has entered saturation, consistent with the expected behavior at higher currents. The subtractor block is implemented using a PMOS-driver operational transconductance amplifier (OTA) configured with resistive feedback. A stable reference level is applied to establish an output common-mode voltage of 1.65 V. As a result, the subtractor output represents the logarithm of division of the gas-sensor and reference-sensor currents, centered about the 1.65 V common-mode point, shown in Figure 7a. The sawtooth waveform generator, shown in Figure 7d, is driven by an active-low reset signal that initiates oscillation. This behavior is verified by the transient response in Figure 8b. The oscillation frequency is tunable through the control resistor *R*_6_, allowing flexible adjustment of the sawtooth period. In this design, selecting *R*_6_ = 13 kΩ produces a stable oscillation frequency of approximately 10.3 kHz. Adjusting resistor *R*_6_ allows direct control over the capacitor charging current, which in turn tunes the sawtooth and PWM frequencies.

The PWM modulation performance is illustrated in Figure 8c. The duty-cycle variation closely follows the divider output, demonstrating high consistency and confirming the correct operation of the modulation stage. Furthermore, the divider output matches the theoretically expected ratio of *I*_gas_/*I*_without gas_, and the expected peaks corresponding to specific *V*_gs_ levels are clearly observable, demonstrating successful translation of sensor current ratios into their PWM-encoded digital representation. This enables robust and accurate interfacing of the sensor output with the analogue-to-digital conversion circuitry, ensuring reliable digital representation of gas-dependent current variations. Furthermore, ensures a stable, noise-resilient interface between the sensor front-end and embedded system, supporting precise real-time gas detection.

The performance of the proposed Ag-rGO–based single-FET multigas sensing platform is benchmarked against previously reported sensors, as summarized in Table 2. The presented approach demonstrates superior capability in simultaneously detecting multiple gases, offering notable advantages in terms of comprehensive cross-gas interference analysis, room temperature operation, and proposes an interfacing circuit.

**Table 2:** Comprehensive Comparison of Reported Multigas Sensing Technologies

**ref**	**no.**	**technique**	**type**	**LOD**	**cross**	**temp**	**voltage**	**circuit**
^21^	4	kinetic analysis over chemiresistive sensor	quant.	25 ppm	no	473 K		no
^22^	6	sensor array + deep learning	quant.	1 ppm	yes	523 K	2.5 V	yes
^23^	7	chemiresistive + potentiometric sensors	quant.	26.6 ppb	no	673 K		no
^24^	3	NDIR spectral method	quant.	500 ppm	no	300 K	–0.75–2 V	no
^25^	1	gate polarity modulation (FET)	qual.	N/A	no	300 K	±30 V	yes
^26^	2	gate-tunable single FET	qual.	N/A	no	300/473 K	±40 V	no
^27^	1	FET for benzene sensing	quant.	500 ppb	yes	300 K	±5 V	no
**this work**	**3**	**single FET platform**	**quant.**	**Ac @ 158 ppb**	**yes**	**300 K**	±**5 V**	**yes**
				**F @ 31 ppb**				
				**Am @ 36 ppb**				

## Conclusion

This work demonstrates a single silver-decorated reduced graphene oxide (Ag–rGO) field-effect transistor (FET) capable of selective detection and discrimination of acetone, formaldehyde, and ammonia through gate-voltage–dependent modulation of surface adsorption phenomena. Distinct sensing signatures observed at gate biases of −4.4, −3.0, and −1.8 V confirm that gate-controlled carrier modulation enables robust discrimination among formaldehyde, acetone, and ammonia, while also facilitating comprehensive cross-sensitivity analysis under mixed-gas conditions. The proposed sensing platform further demonstrates low-concentration detection capability, with estimated limits of detection (LOD) of 158 ppb for acetone, 31 ppb for formaldehyde, and 36 ppb for ammonia, highlighting its strong potential for selective room-temperature multigas sensing applications. This validates the potential of a single Ag–rGO FET to replace traditional multisensor arrays. Furthermore, a 180 nm CMOS readout circuit architecture, comprising an analogue front-end, has been designed and simulated to demonstrate the feasibility of integration with the proposed FET gas sensor, thereby enabling the development of a compact and low-power gas sensing system.

## Supplementary Material



## Notes

Ag–rGO synthesis and preparation schematic; multigas selectivity; dynamic response; transient adsorption/desorption characteristics; statistical distribution of sensor response; limit-of-detection calculations; gas-sensing measurement setup and experimental procedure (PDF)
