# Supplementary material for: Gate-Tunable Single Ag–rGO FET Platform for Room-Temperature Trigas Detection with a CMOS-Compatible PWM Readout Interface
Source: ACS Omega. 2026 Oct 10;11(41):63075–83. doi: 10.1021/acsomega.6c05753 (PMC13689154; doi:10.1021/acsomega.6c05753)
Supplement: Supplementary file 1 [file ao6c05753_si_001.pdf]

Gate-Tunable Single Ag–rGO FET Platform for Room-Temperature Tri-Gas Detection with CMOS-Compatible PWM Readout Interface

Mayank Kohli[1], Nimisha[2], Swapnil Sharma[1], Santanab Majumder[1], Gourab Dutta[1], and Tarun Kanti Bhattacharyya*[1]

[1]Department of Electronics and Electrical Communication Engineering, Indian Institute of Technology, Kharagpur, 721302, India.
[2]Manipal School of Information Sciences, Manipal, 576104, India.

*Email: tkb@ece.iitkgp.ac.in

Supplementary Information

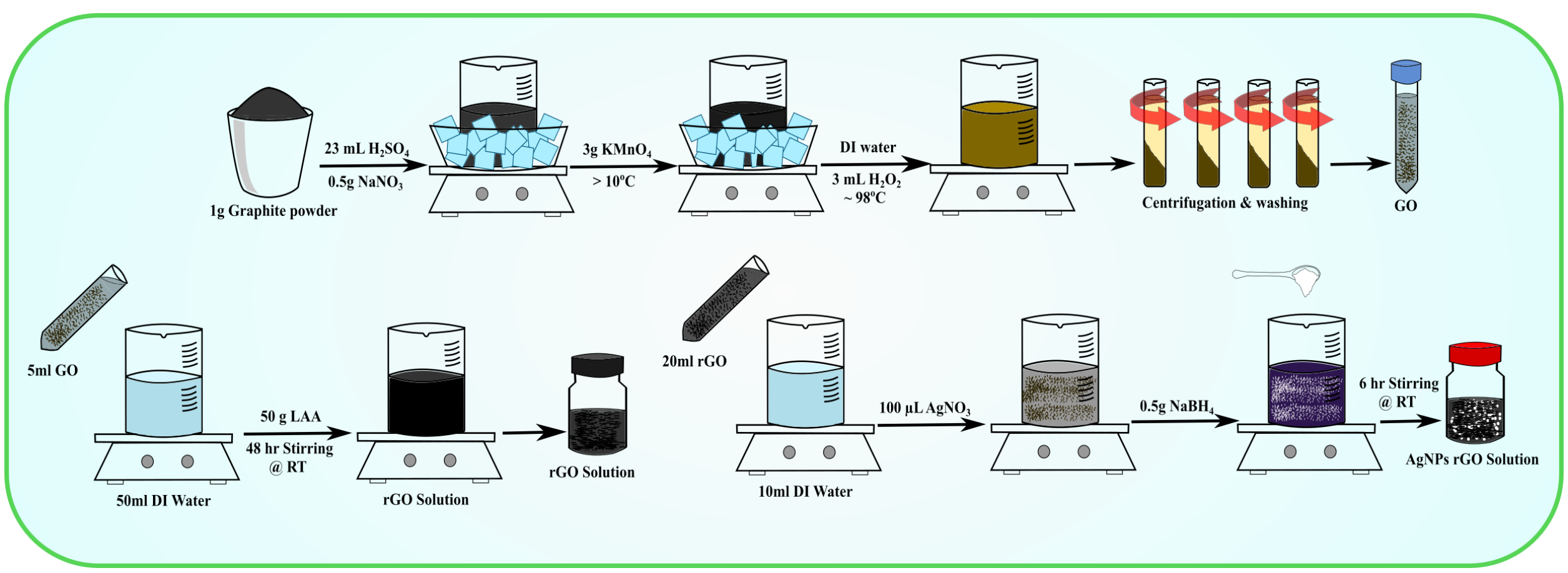

Figure S1: Step-by-step schematic representation of the synthesis process involving oxidation of graphite to Graphene Oxide (GO), subsequent reduction to obtain rGO followed by surface decoration with silver nanoparticles to form Ag–rGO.

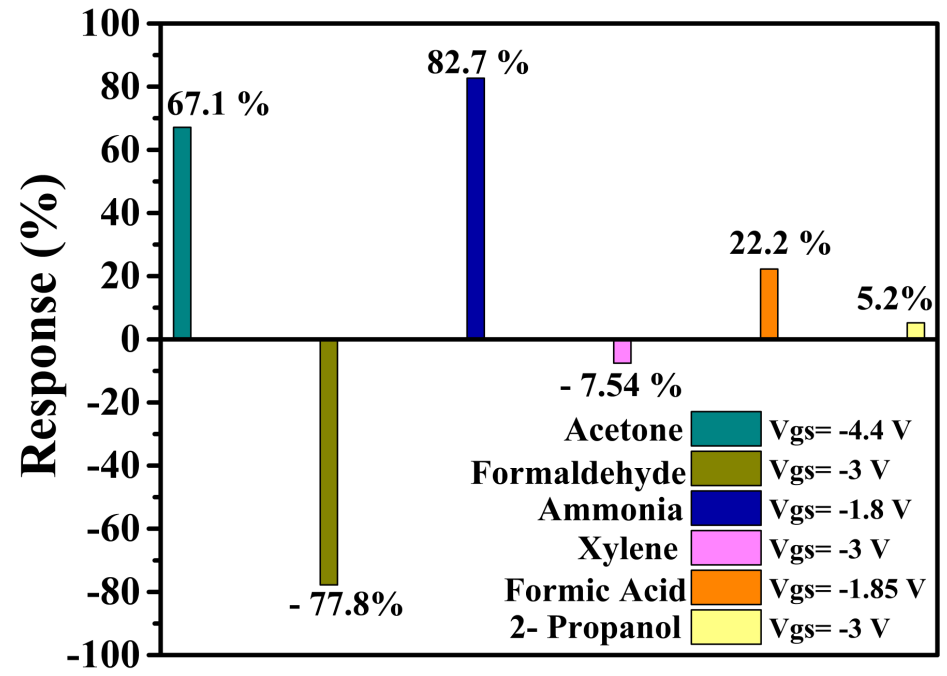

Figure S2: Selectivity assessment of the Ag–rGO FET sensor toward multiple volatile organic compounds (VOCs).

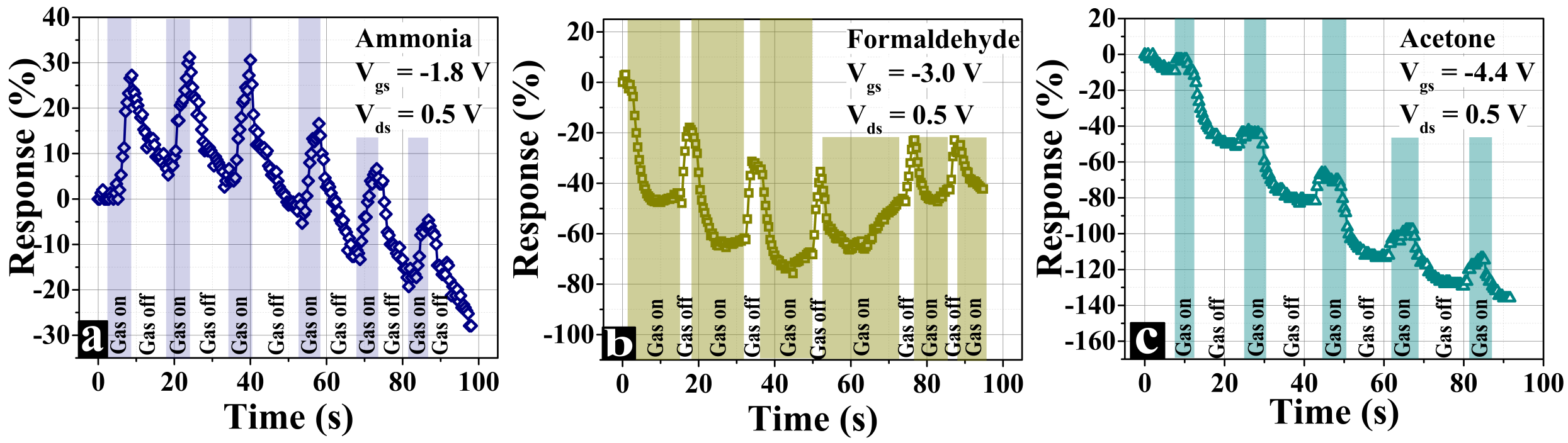

Figure S3: Cyclic dynamic response of the Ag–rGO FET sensor upon repeated exposure to (a) ammonia, (b) formaldehyde, and (c) acetone at 10 sccm.

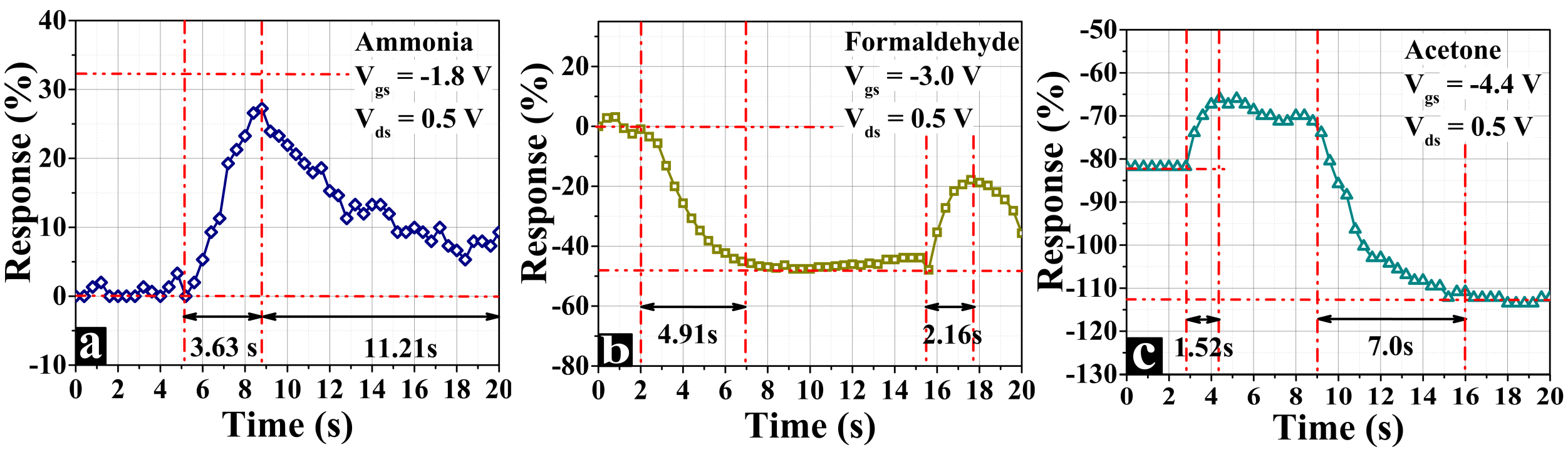

Figure S4: Transient response analysis showing adsorption and desorption kinetics of the Ag–rGO FET sensor for (a) ammonia, (b) formaldehyde, and (c) acetone.

Table 1: Limit of detection (LOD) of the gas sensor calculated using the 3σ method.

Analyte	σ (%)	3σ (%)	Sensitivity (% ppm^{-1})	LOD (ppm)
Acetone (-4.4 V)	1.26	3.78	24	0.158
Formaldehyde (-3.0 V)	0.40	1.20	38.8	0.031
Ammonia (-1.8 V)	0.17	0.51	14.0	0.036

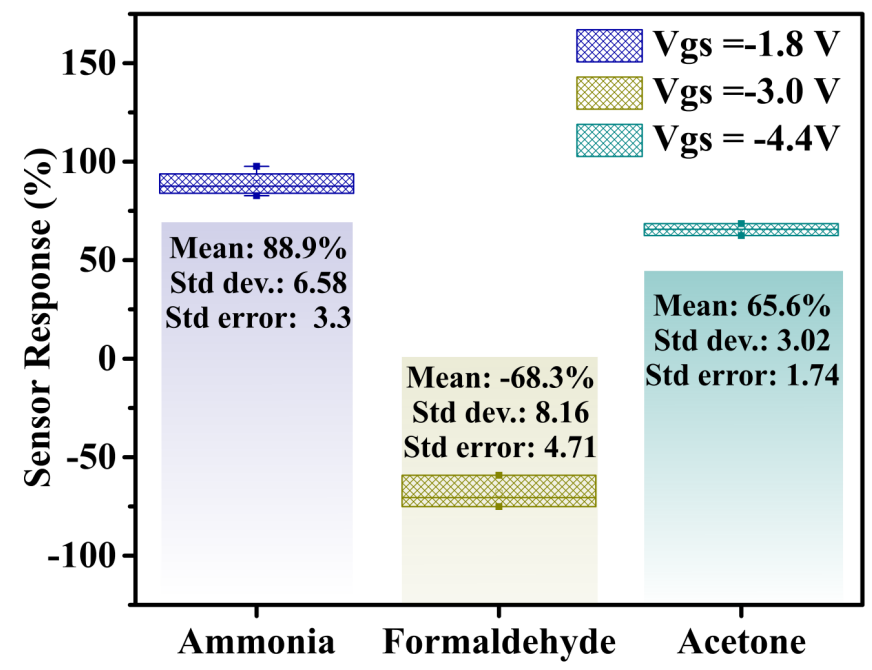

Figure S5: Statistical distribution of the Ag–rGO FET sensor response toward different analytes.

LOD Calculation

The limit of detection (LOD) of the fabricated sensor was estimated using the 3σ criterion, where the LOD is defined as ($LOD = 3\sigma/S$). Here, (σ) represents the standard deviation of the blank response (ambient environment) obtained from five independent measurements under identical experimental conditions, and (S) is the sensitivity (slope) of the calibration curve expressed in ($\%ppm^{-1}$). The blank responses exhibited standard deviations of 1.26%, 0.40%, and 0.17% for acetone, formaldehyde, and ammonia (at -4.4V, -3.0V and -1.8V), respectively. Based on the corresponding calibration slopes (given in Fig.5(c)) of 24.0, 14.0, and -38.8 (% ppm^{-1}), the estimated LODs were 158 ppb for acetone, 31 ppb for formaldehyde, and 36 ppb for ammonia.

Gas Sensing Measuring Setup

Gas sensing measurements were performed in a custom-built airtight chamber with an internal volume of 10 L at room temperature. Dry air was used as the carrier gas to establish the baseline and to purge the chamber after each sensing cycle. Certified gas cylinders containing 1000 ppm of the target analytes served as the source gases. The desired gas concentrations were generated by dynamic dilution with dry air using calibrated alicat mass flow controllers (MFCs). The gas mixture was introduced into the sensing chamber while maintaining a constant total flow rate. Each sensing cycle consisted of 30 s of gas exposure, followed by recovery under dry air until the baseline current was restored.

The fabricated FET sensor was electrically connected to a Keysight B1500A Semiconductor Device Analyzer, which was used to apply the required gate and drain biases and to continuously record the drain current (I_{ds}) during the sensing and recovery processes. The sensor response was determined from the relative change in drain current with respect to the baseline current recorded under dry air.
